# Correlation between changes in apathy and cognition in Alzheimer’s disease associated apathy: Analysis of the Apathy in Dementia Methylphenidate Trial 2 (ADMET 2)^☆^

**DOI:** 10.1016/j.inpsyc.2024.100012

**Published:** 2024-12-04

**Authors:** Krushnaa Sankhe, Shankar Tumati, Jamie Perin, Luc Rivet, Danielle Vieira, Paul B. Rosenberg, Nathan Herrmann, David Shade, Alan J. Lerner, Prasad R. Padala, Olga Brawman-Mintzer, Christopher H. van Dyck, Anton P. Porsteinsson, Suzanne Craft, Allan I. Levey, Jacobo Mintzer, Krista L. Lanctôt

**Affiliations:** a Sunnybrook Research Institute, University of Toronto, Toronto, ON, Canada; b Johns Hopkins University, Bloomberg School of Public Health, Baltimore, MD, USA; c Johns Hopkins University, Department of Medicine, Baltimore, MD, USA; d University Hospitals Cleveland Medical Center, Case Western Reserve University School of Medicine, Cleveland, OH, USA; e Central Arkansas Veterans Healthcare System, University of Arkansas for Medical Sciences, Little Rock, AR, USA; f Ralph H. Johnson VA Medical Center, Medical University of South Carolina, Charleston, SC, USA; g Yale School of Medicine, New Haven, CT, USA; h University of Rochester School of Medicine and Dentistry, Rochester, NY, USA; i Wake Forest University, Winston-Salem, NC, USA; j Emory Goizueta Alzheimer’s Disease Research Center, Atlanta, GA, USA; k Department of Psychiatry, University of Toronto, Toronto, ON, Canada

**Keywords:** Apathy, Alzheimer’s disease, Cognition, Methylphenidate

## Abstract

**Background:**

Previous trials have shown improvements in both apathy and cognition with methylphenidate (MPH).

**Objectives:**

To assess whether changes in apathy correlated with changes in cognition in the Apathy in Dementia Methylphenidate Trial 2 (ADMET 2).

**Participants:**

Mild to moderate AD patients with clinically significant apathy randomized to MPH (20 mg/day) or placebo for 6 months.

**Measurements:**

Apathy was measured with the Neuropsychiatric Inventory-apathy (NPI-A) domain. Cognition was measured using the Mini-Mental State Exam (MMSE), Hopkins Verbal Learning (immediate [HVLT-I], delayed [HVLT-D] recall), Digit Span (Forward [DF], Backward [DB]), Trail Making (TMT-A, TMT-B), Action Verbal Fluency (AV), Category Fluency (CF), and the Short Boston Naming Test (BNT).

**Design:**

Linear mixed models included cognitive change scores as dependent variables and time, treatment, change in NPI-A and the interaction between treatment and change in NPI-A as independent variables, which were additionally adjusted for baseline NPI-A and cognitive scores, age, sex, level of education and presence of diabetes.

**Results:**

199 participants (66 % male) were included (98-MPH, 101-placebo). Among all participants, worsening CF was associated with worsening apathy (−0.15 (0.05), p = .003). In addition, change in HVLT-I was associated with the interaction between changes in apathy and treatment (−0.31 (0.07), p = 0.0000158).

**Conclusion:**

Changes in apathy are mostly independent of cognitive changes and apathy response to MPH may be independent from cognition. These results are consistent with the view that apathy as a syndrome is related to but distinct from cognition.

## Introduction

Neuropsychiatric symptoms (NPS) are non-cognitive behavioral symptoms that are core features of Alzheimer’s disease (AD) and related dementias [31,54]. A meta-analysis of 48 studies in AD revealed that apathy was the most frequent NPS with an overall prevalence of 49 % [54], and ranges from 24–85 % in AD dementia [28]. Recent diagnostic criteria for apathy in neurocognitive disorders have defined apathy as reduced goal-directed behavior with symptoms in at least two of its three dimensions: diminished initiative, diminished interest, and diminished emotional expression [35]. Apathy symptoms have been linked to a host of negative outcomes such as decreased quality of life [18], increased mortality [39], increased care needs [14], higher caregiver burden [21], increased risk of institutionalization [1] and higher costs of care [16,24]. In those with Mild Cognitive Impairment, apathy is a key predictor of progression to AD dementia [42,47]. Apathy has also been associated with greater cognitive and functional decline in those with AD. For example, in a longitudinal study of 354 AD patients with 1- and 4-year follow-up after baseline in 70 % of the patients, baseline apathy was associated with faster functional and cognitive decline measured by the Functional Independence Measure score and the Mini-Mental State Examination (MMSE) [47].

Cross-sectionally, apathy has consistently been associated with cognitive impairment where apathetic AD patients have demonstrated more impairment in attention [34], visual memory [15], verbal memory [25,34], and verbal fluency [10,25], than patients without apathy. Among the cognitive domains, executive dysfunction was the best predictor of apathy in AD, especially lack of initiative [11]. Previous randomized placebo-controlled trials showed improvements in both apathy and cognition with methylphenidate (MPH) [27,41,45]. MPH is a psychostimulant that acts on dopaminergic and norepinephrine systems in the central nervous system. It increases synaptic dopamine and norepinephrine in the brain by blocking the dopamine and norepinephrine transporters on the presynaptic neuron [12]. Together, dopamine and norepinephrine influence different components of goal-directed behavior (e.g. motivation and energy levels) [50,51]. In a group of community-dwelling veterans with mild AD, MPH improved apathy as well as cognition [41], particularly global cognition measured by the MMSE. The Apathy in Dementia Methylphenidate Trial (ADMET), a 6-week randomized controlled trial comparing participants taking placebo (PLB) medication or MPH for apathy in AD, showed a trending improvement in global cognition (measured by MMSE) [45], and improved selective attention (measured by digit span forward) [27].

As apathy has previously been associated with cognitive impairment, particularly executive functions (EF) such as selective attention, the nosological position of apathy as a separate syndrome from cognition may be debated. Among pharmacological agents, MPH has been shown to improve both apathy and cognition, which can present a challenge to discern whether these changes occur independently of each other. If impaired cognition drives apathy, drug treatments targeting cognitive impairment should also improve apathy, presenting a regulatory challenge. Similarly, improvement in apathy should be accompanied by improvements in cognition. To overcome this challenge, it is important to determine whether MPH, which can also improve attention, improves apathy independent of cognition. The Apathy in Dementia Methylphenidate Trial 2 (ADMET 2) is the largest randomized, double-blind, placebo-controlled, 6-month, multi-center, phase 3 clinical trial investigating the efficacy of MPH for apathy in patients with mild to moderate AD [36]. ADMET 2 found MPH treatment resulted in improvement in apathy (with a small to medium effect size) and was well-tolerated [36]. Among other NPS, there were no changes in depression, anxiety or other NPS as determined on the Neuropsychiatric Inventory (NPI) from baseline to 6-month follow-up [6].

In this secondary analysis, we aim to assess whether changes in apathy are associated with changes in various cognitive domains in treated patients. To address this aim we used data from ADMET 2. We hypothesize that there will be no significant associations between changes in apathy and changes in global cognition and in individual cognitive domains among participants in ADMET 2.

## Methods

In ADMET 2, 200 participants with mild to moderate AD and clinically significant apathy were randomized to receive 20 mg/day of methylphenidate or placebo in a 1:1 ratio for 6 months. All caregivers also received a standardized psychosocial intervention at monthly study visits.

### Informed consent and ethics approval

ADMET 2 was conducted in accordance with the Declaration of Helsinki. Both study participant and caregiver were required to provide informed consent prior to study entry and administration of treatment intervention. The Institutional review board (IRB) of the Johns Hopkins Bloomberg School of Public Health reviewed and approved the protocol on 9 July 2015 IRB number 00006303. The individual clinical centres also received approval from their respective IRBs; details have been listed elsewhere [46].

### Participants

The full inclusion and exclusion criteria have been detailed elsewhere [46] and are briefly summarized here. Eligible participants had a diagnosis of possible or probable AD based on the National Institute of Neurological and Communicative Disorders and Stroke – Alzheimer’s Disease and Related Disorders Association with a MMSE score of 10–28; clinically significant apathy assessed by the Neuropsychiatric Inventory – apathy (NPI-A) with a frequency × severity score of 4 or greater; a medication for apathy was deemed appropriate; availability of a caregiver who spent greater than ten hours a week with the participant; sufficient fluency of written and spoken English; if female, post-menopausal.

Participants were excluded if they met criteria for a Major Depressive Episode by the Diagnostic Statistical Manual of Mental Disorders – IV (TR) criteria; clinically significant agitation/aggression, delusions, hallucinations on the NPI; change to AD medications within 30 days of randomization; change in antidepressant within 30 days of randomization or 5 half-lives; use of trazodone > 50 mg or lorazepam > 0.5 mg for indications other than sleeping difficulties within 30 days of randomization or 5 half-lives; taking other benzodiazepines; failure of treatment with methylphenidate in the past; currently taking an amphetamine, antipsychotic, bupropion, monoamine oxidase inhibitors, tricyclic antidepressant within 30 days of randomization or 5 half-lives; need for acute psychiatric hospitalization or suicidal; significant communicative impairments; central nervous system abnormalities, seizures, Tourette’s syndrome, presence of motor tics, abnormal electroencephalogram results; lack of appetite that results in significant unintentional weight loss; uncontrolled hyperthyroidism; any cardiovascular or cerebrovascular abnormality deemed to be clinically significant, tachycardia, uncontrolled hypertension; closed angle glaucoma or pheochromocytoma; women with childbearing potential; current participation in a clinical trial; any condition that made it medically inappropriate or risky for participants.

### Measures

In ADMET 2, apathy was measured using the NPI-A domain collected at baseline and subsequent monthly visits. The NPI is a structured interview with the caregiver and assesses the frequency and severity of twelve NPS [7]. The score for each NPS is calculated by multiplying frequency (scored from 1–4) and severity (scored from 1–3) of the symptom. Caregiver distress is also scored from 1 to 5. Higher scores indicate greater frequency, severity, and distress.

Cognitive outcome measures were assessed at baseline and every two months (follow-up visits at 2, 4, and 6 months). These included the Mini-Mental State Exam (MMSE), Hopkins Verbal Learning Test – Revised (HVLT-R), the Digit Span: The Wechsler Adult Intelligence Scale – Revised Digit Span sub-test-Digit Forward (DF) and Digit Backward (DB), Trail Making Tests (TMT-A and TMT-B), Action Verbal Fluency Test (AV) from the Parkinson’s Disease–Cognitive Rating Scale, Category Fluency Task-Animal Naming (CF), and the Short Boston Naming Test (BNT) [46]. The MMSE measures general cognition and is administered to the participant. Scores range from 0 to 30 with higher scores indicating better cognitive function [13]. The MMSE was the major cognitive outcome for ADMET 2 [36] and MPH has been shown to improve the MMSE previously [41,45]. The HVLT-R measures verbal learning, recognition, and delayed recall. This test consists of immediate recall (HVLT-I) and delayed recall (HVLT-D). Scores range from 0–12 with higher scores indicating better performance [46]. The Digit Span (DS) measures auditory attention and working memory [22]. Separate scores are obtained for spans read forward and backward ranging from 0–9. For DF, digit sequences of progressively increasing length were read by the examiner and patients were instructed to repeat them in the same order. In the DB condition, patients were required to repeat the digits in the reverse order. One point was given for each correct sequence and the test was terminated when patients were unsuccessful on two trials of the same sequence length. Performance was summarized by DS forward (selective attention) and DS backward (working memory) [27]. Higher scores indicate higher cognitive function. TMT-A and TMT-B measure attention, EF, and visuo-motor tracking [46]. They are scored by the time taken to complete the test, where a shorter time (measured in seconds) is indicative of higher cognitive function [46]. The AV fluency test measures EF, working memory and information processing speed. The score is calculated by the total number of unique verbs with higher counts indicating less cognitive impairment [46]. The CF task measures EF, working memory, set shifting and executive control. In this task, participants are asked to verbalize animal names, where a higher count indicates less cognitive impairment [46]. Lastly, the BNT test measures visual confrontational naming. Scores range from 0–15 with higher scores indicating better control of expressive language [46].

### Statistical analysis

This was a secondary analysis of ADMET 2. Demographics and baseline characteristics were compared between MPH and PLB groups using t-tests for continuous data and chi-squared tests for categorical data.

A linear mixed model was used to calculate changes in apathy over time. This model included changes in apathy as the dependent variable and time, age, sex, diabetes, level of education, baseline apathy and treatment group as covariates.

To assess the relationship between changes in apathy and cognition among all participants, a linear mixed model was used with cognitive change scores as dependent variable and time, treatment, change in apathy, and the interaction between treatment and change in apathy as independent variables in unadjusted models. Adjusted models additionally included the following covariates: baseline cognitive test score, baseline NPI-A, age (in tertiles), sex, level of education, and presence of diabetes. This model was also used to assess changes in cognition over time in all participants.

If an interaction effect was found, the cognitive change scores was assessed in each treatment group separately, with the cognitive change score as the dependent variable, and time and change in NPI-A score as independent variables. Adjusted models included the following covariates: baseline cognitive test score, baseline NPI-A, age (in tertiles), sex, level of education and presence of diabetes.

Change scores for NPI-A and cognitive scores were calculated as the difference between each time point and baseline (6-month follow up – baseline, 4-month follow up – baseline, 2-month follow up – baseline). For all models, significance was set at a two-sided p-value of < 0.05 with Bonferroni correction for multiple comparisons [10 cognitive tests p < 0.005]. Mean change in cognitive scores at 2, 4, and 6- month follow-up by treatment group were also calculated (Supplementary table 1).

## Results

In this analysis, 199 participants (131 (66 %) male) were included, with 98 participants randomized to the MPH group and 101 in the PLB group (Table 1). Participant demographics and characteristics at baseline were comparable between the treatment arms, except on presence of diabetes. The change in NPI-A in the whole sample at each time point are as follows: 2-month follow up (B(SE) = −2.83 (0.25), p < 0.001), 4 month follow up (B(SE) = −2.78 (0.26), p < 0.001), and 6-month follow up (B(SE) = −2.77 (0.26), p < 0.001).

### Overall Group (Table 2).

Among all participants, worsening CF scores were associated with worsening NPI-A (**B(SE) = −0.15 (0.05), p = 0.003),** after adjusting for multiple comparisons (p < 0.005). There was no significant interaction effect between changes in apathy and treatment group on CF scores (**B(SE) = 0.04 (0.07), p = 0.58).** No associations were found between change scores on other cognitive tests and change on NPI-A.

Among all cognitive change scores, the interaction term (change in apathy × treatment group) was significantly associated only with the HVLT-I **(B(SE) = −0.31 (0.07), p = 0.000016).** Subsequent analysis found that HVLT-I change scores were associated with changes in apathy in the placebo subgroup **(B(SE) = −0.30 (0.06), p = 0.0000017),** but not in the MPH subgroup **(B(SE) = 0.08 (0.06), p = 0.19).**

Among all participants, MMSE scores worsened at all time points (2, 4, 6-month follow-up). CF scores worsened at 4-month and 6-month follow up, HVLT-I worsened at 2-month follow up, and BNT worsened at 6-month follow up (Supplementary table 2). HVLT-I scores did not significantly decline over time in the MPH group and worsened at the 2-month and 6-month follow up in the PLB group (Supplementary table 3).

## Discussion

This study evaluated whether changes in apathy were associated with changes in cognition, adjusting for baseline measures among apathetic AD patients in the ADMET 2 trial. Among all participants, changes in apathy were associated with only one of four tests of EF, and not with other cognitive domains, and this change did not differ between treatment groups. The clinical meaningfulness of an isolated change on one test of executive function may be considered relatively small. The change in apathy with treatment group interaction was also not significant for all other cognitive tests, except in the model with verbal learning immediate recall (HVLT-I).

In this analysis, changes in apathy were not associated with changes in global cognition, as measured by the MMSE among all participants or by treatment group. This finding is consistent with those reported for ADMET 2 [36], where the largest change in apathy occurred during the first 2 months of MPH treatment, which was sustained for 6 months. However, cognition did not follow the same time course, as shown in this analysis, with worsening scores over time and without differences between treatment groups over 6 months [36]. To date, there have been 3 placebo-controlled trials of MPH for the treatment of apathy in AD. While in all 3 studies, apathy improvement after 2 weeks [17], 6 weeks [45] and 12 weeks [41] favoured MPH treatment, the impacts on global cognition were inconsistent. As measured by the MMSE Herrmann et al. [17], did not find improvements in global cognition after 2 weeks of MPH treatment whereas Rosenberg et al. [45] showed trending improvement (1.5 points; 95 % CI = −0.1 to 3.1; p = .06) favoring MPH treatment after 6 weeks. Padala et al. [41] did not find significant group differences on MMSE after 4 weeks; however, they found that the MPH group had significantly greater improvement in MMSE after 12 weeks of MPH treatment. Taken together, those studies suggest that changes in global cognition do not correlate with change in apathy after MPH, but group differences may gradually emerge with detectable differences after 12 weeks. The analysis done here supports those findings by suggesting that the improvement in apathy was not associated with improvements in global cognition.

Among all participants, changes in apathy were not associated with changes in verbal memory (immediate and delayed recall). However, there was an interaction effect between NPI-A and treatment group on immediate recall, indicating that the relationship between changes in apathy and change in verbal learning-immediate recall scores differs depending on the treatment group. In the MPH group, immediate-recall verbal memory scores did not significantly decline over time and no associations with apathy were observed, suggesting that changes in apathy were independent of changes in verbal learning. In the PLB group, immediate recall worsened at the 2-month and 6-month follow-up and was associated with worsening apathy. Patients with AD and apathy have more impairment in impulse control, attention, visual memory, verbal memory, naming, phonological verbal fluency, and semantic verbal fluency than those without apathy [4]. In a cross-sectional study of 124 AD patients, apathy was correlated with poorer global cognition (measured by MMSE), verbal memory (measured by auditory verbal learning test which included immediate recall, short-delayed recall), verbal fluency (measured by animal fluency test) and instrumental activities of daily living (IADL) [52]. Furthermore, only verbal fluency and IADL were independently associated with apathy [52]. A review of 56 studies of healthy volunteers found that MPH improves cognitive performance in older adults in processing speed, verbal learning and memory, and attention and vigilance [30]. However, the same was not found in the current analysis and could be because MPH and its actions on verbal memory domains could differ based on the patients’ severity of cognitive impairment. As scores at each visit did not significantly decline from baseline in the MPH group, MPH could have prevented worsening of performance within the verbal memory domain. More longitudinal research into the mechanism of MPH in the verbal memory domain in the AD population needs to be conducted to confirm these findings.

Among the 4 tests of executive function – CF, DF, TMT-B and AV fluency, changes in apathy were only associated with changes in CF, and no interaction effect between changes in apathy and treatment group on CF scores was found, indicating that the association between changes in NPI-A and CF did not differ between PLB and MPH groups.

Previously, apathy has been associated with executive dysfunction, especially in areas of selective and sustained attention, even after correcting for effects of MMSE [34]. Associations between apathy and selective attention have been studied where apathetic patients have shown reduced brain activity in areas associated with attention [26]. MPH, a drug approved for Attention-Deficit Hyperactivity Disorder (ADHD), has been known to have attention-enhancing effects [9]. Although MPH in younger populations was found to enhance cognition with improvement in academic performance [33], working memory and sustained attention [49], the cognitive effects of MPH in older patients is less established. In a randomized double-blind study of older male volunteers, no significant effects of MPH on working memory, response inhibition or sustained attention were seen when compared to younger participants [49]. Animal studies of MPH on attention showed that task performance was improved in adult rats but not in aged rats [3]. They suggested that MPH was unable to play its cognitive enhancing effects in aged rats due to the decline of noradrenergic projections from the locus coeruleus to the prefrontal cortex that occur with normal aging [3]. AD patients consistently display lower levels of dopamine and dopamine receptors [37,43] and a loss of neurons in the noradrenergic locus coeruleus [5] when compared to controls. Therefore, this could suggest that target areas for MPH’s mode of action for its cognitive effects may be less available in this population. In the ADMET trial, improvements in selective attention measured by DF scores favored those on MPH after 6 weeks of treatment. Response prediction analyses found that attention and apathy may respond independently to MPH [27]. In the current analysis, however, no associations were observed between changes in apathy and changes in DF. The inconsistent results may be due to both executive dysfunction and hypofrontality playing a role in the pathophysiological mechanism of apathy [52]. Taken together, in this analysis, only one of the tests of EF, CF, showed that its changes were associated with apathy. These findings suggest that sub-types of EF may differ in their association with apathy.

It is important to assess the clinical meaningfulness of results found in this study. Among all participants, for each one unit improvement in apathy on the NPI apathy domain over 6 months, CF, as measured by animal naming, improved by 0.15 units. In AD patients, animal naming scores decrease by 0.74 units annually [53]. Assuming that such a change occurs in a linear fashion, over the 6-month duration of ADMET 2, a decline of 0.37 units can be expected. Over 6 months of ADMET 2, CF scores declined by a greater extent in both treatment arms. Hence, the 0.15 unit improvement in CF scores associated with a 1 point improvement in NPI-A appears to be substantial. However, since there were no associations between changes in apathy and the other EF tests (DF, TMT-B and AV fluency), the clinical meaningfulness of a change on one test of EF (CF) may be considered relatively small.

The lack of association between changes in apathy and the cognitive domains measured in this analysis suggest that apathy and cognition may have different neural correlates. Neuroimaging correlates of apathy suggest frontal-subcortical networks are involved in the occurrence of apathy in AD [48]. More specifically, altered functional and structural properties in areas of the anterior cingulate cortex (ACC) and orbitofrontal cortex (OFC) have been identified; these areas are associated with initiation and interest respectively [29,48]. Higher apathy scores in AD patients were associated with increased tau deposition in the OFC, decreased OFC thickness and decreased fractional anisotropy in the uncinate fasciculus (UNC) (structurally connected to OFC), with no association with amyloid-β (Aβ) pathology [23]. In that study, no correlations were found between apathy scores and cognitive scores (measured by MMSE) and between MMSE scores and tau deposition in the OFC [23]. The results suggest that tau deposition in the OFC was not directly associated with cognition but may cause functional deficits in the OFC-UNC network resulting in apathy [23]. Although that cohort consisted of patients with early-stage AD, their MMSE scores were similar to that in the ADMET 2 cohort. Another study found a positive correlation between NPI-A severity and Aβ (measured by positron emission tomography) in medial and orbitofrontal areas, insula, and right ACC in 28 AD patients [38]. Correlations between MMSE scores and Aβ deposition were not conducted in that study [38]. However, among cognitively unimpaired older adults, baseline cerebrospinal fluid Aβ pathology was associated with increasing apathy severity over time, which was independent of cognitive changes [19] measured by the modified Preclinical Alzheimer’s Cognitive Composite (a composite score that included the MMSE, Logical Memory Delayed Story Recall, the Digit-Symbol Substitution Test, and recall from the Free and Cued Selective Reminding Test) [44]. For dopaminergic signalling, a PET imaging study found that due to the age-related decline in dopamine signalling in the brain, sensitivity to MPH in areas of the brain associated with reward regions may also be affected [32]. This may also impact the relationship between apathy and cognition over time. Future studies in this area could aim to include neuroimaging and path analysis models to further evaluate the independent neurobiological mechanisms underlying apathy and cognitive changes, particularly when taking pharmacological compounds like MPH.

To conclude, here we found that across all participants, changes in apathy were associated with changes in category fluency, a measure of executive function, and this association did not differ between treatment groups. Among all participants, changes in apathy were not associated with changes in global cognition, verbal memory, processing speed, working memory, or expressive language in participants with mild to moderate AD. A strength of the current study was adjusting for baseline cognition and apathy in all analyses. A limitation of this study was that no neuroimaging biomarkers were available to assess the correlation of apathy with cognitive performance. Future work could investigate neuroimaging correlates to understand the pathways of cognitive change, especially in measures of EF associated with apathy. Furthermore, ADMET2 used the NPI as the primary outcome to assess apathy. While the NPI is a widely used tool to assess neuropsychiatric symptoms in neurodegenerative disorders and has robust psychometric properties [8], more comprehensive apathy scales may find different results. Another limitation was the use of the MMSE as a global cognitive measure. Although the MMSE is widely used for staging and documenting cognitive change over time [2], it may fail to identify executive dysfunction [20,40]. Future studies assessing global cognition using additional approaches would help bolster these findings. Despite these limitations, our findings provide evidence that changes in apathy are mostly independent of changes in cognition. Overall, these findings suggest that apathy responds to MPH independently from cognition and these results are consistent with the view that apathy as a syndrome is related to but distinct from cognition.

## Supplementary Material

Supplementary Table 1

Supplementary Table 2

Supplementary Table 3

## Figures and Tables

**Table 1:** Baseline characteristics of ADMET 2 participants.

	Overall (n = 199)	MPH (n = 98)	PLB (n = 101)	t/ χ^2^ (df), p
**Age, n (%)**				
52 −72 yrs	66 (33.0)	28 (28.6)	38 (37.6)	
73 −79 yrs	62 (31.0)	35 (35.4)	27 (26.7)	2.52 (2), 0.28
> 80 yrs	71 (35.5)	35 (35.4)	36 (35.6)	
**Sex, n (%)**				
Male	131 (65.5)	65 (65.7)	66 (65.3)	0.003 (1), 0.96
Female	68 (34.0)	33 (33.3)	35 (34.7)	
**Diabetes, n (%)**	31 (15.5)	10 (10.1)	21 (20.8)	4.24 (1), 0.04 *
**Education, n (%)**				
HS or less	49 (24.5)	24 (24.2)	25 (24.8)	1.04 (3), 0.79
Some College	40 (20.0)	17 (17.2)	23 (22.8)	
Bachelor’s Degree	65 (32.5)	34 (34.3)	31 (30.7)	
Graduate/Professional	45 (22.5)	23 (23.2)	22 (21.8)	
**Medications, n (%)**				
Memantine	75 (37.5)	38 (38.4)	37 (36.6)	0.097 (1), 0.76
Cholinesterase Inhibitors	145 (72.5)	75 (75.8)	70 (69.3)	1.31 (1), 0.25
SSRI	71 (35.5)	32 (32.3)	39 (38.6)	0.78 (1), 0.38
SNRI	13 (6.5)	7 (7.1)	6 (5.9)	0.12 (1), 0.73
Any AD Medications	157 (78.5)	79 (79.8)	78 (77.2)	0.34 (1), 0.56
**Baseline blood pressure, mean (SD)**				
Systolic	136.7 (17.5)	135.8 (18.2)	137.5 (16.8)	−0.64 (196), 0.52
Diastolic	76.1 (9.7)	76.2 (9.5)	75.9 (9.88)	0.21 (196), 0.84
**Cognitive Tests, mean (SD)**				
MMSE	18.93 (4.82)	19.18 (4.75)	18.68 (4.90)	0.73 (198), 0.47
CF	9.31 (5.18)	9.65 (5.59)	8.98 (4.74)	0.91 (190), 0.36
HVLT-I	10.01 (5.54)	9.82 (5.17)	10.19 (5.89)	−0.46 (193), 0.64
HVLT-D	0.83 (1.80)	0.78 (1.57)	0.88 (2.00)	−0.38 (185), 0.71
AV	8.85 (4.34)	8.65 (4.44)	9.05 (4.26)	−0.64 (195), 0.52
BNT	10.55 (3.77)	10.78 (3.63)	10.33 (3.91)	0.83 (195), 0.41
DF	8.16 (2.25)	8.21 (2.16)	8.11 (2.36)	0.30 (194), 0.77
DB	5.31 (2.55)	5.14 (2.58)	5.47 (2.53)	−0.89 (195), 0.37
TMT-A	85.68 (56.68)	80.56 (46.45)	90.67 (65.04)	−1.15 (149), 0.26
TMT-B	163.46 (60.89)	170.24 (50.94)	157.06 (69.11)	0.89 (62), 0.37

Abbreviations: MPH, methylphenidate; PLB, placebo; AD, Alzheimer’s Disease; SSRI, selective serotonin reuptake inhibitors; SNRI, serotonin and norepinephrine reuptake inhibitors; NPI, neuropsychiatric inventory; MMSE, Mini Mental State Examination; CF, Category Fluency; HVLT-I, Hopkins Verbal Learning-immediate recall; HVLT-D, Hopkins Verbal Learning-delayed recall; AV, Action Verbal Fluency; BNT, Boston Naming Test; DF, Digit forward span; DB, Digit backward span; TMT-A, Trail Making Test-A; TMT-B, Trail Making Test-B; HS, high school; df, degrees of freedom.

* p < 0.05

**Table 2:** The association between change in cognition and change in NPI-apathy among all participants (n = 199) on linear mixed models^#^.

Cognitive Test		All participants (n = 199)			
		Unstandardized coefficients		T value	Pr (> |t|)
		B	Std. Error		
**MMSE**	Unadjusted	−0.09	0.04	−2.09	0.04
	Adjusted	−0.09	0.04	−1.94	0.05
**CF**	Unadjusted	−0.15	0.05	−3.13	0.002 * **
	Adjusted	−0.15	0.05	−2.98	0.003 * **
**HVLT-I**	Unadjusted	0.04	0.06	0.70	0.49
	Adjusted	0.03	0.06	0.48	0.63
**HVLT-D**	Unadjusted	0.01	0.02	0.68	0.50
	Adjusted	0.02	0.02	0.89	0.38
**AV**	Unadjusted	0.08	0.05	1.62	0.11
	Adjusted	0.08	0.05	1.50	0.14
**BNT**	Unadjusted	−0.009	0.03	−0.28	0.78
	Adjusted	−0.006	0.03	−0.19	0.85
**DF**	Unadjusted	−0.003	0.03	−0.10	0.92
	Adjusted	0.002	0.027	0.07	0.94
**DB**	Unadjusted	−0.04	0.03	−1.34	0.18
	Adjusted	−0.04	0.03	−1.30	0.19
**TMT-A**	Unadjusted	−0.36	0.77	−0.47	0.64
	Adjusted	−0.02	0.83	−0.03	0.98
**TMT-B**	Unadjusted	−0.72	1.30	−0.56	0.58
	Adjusted	−0.11	1.33	−0.08	0.94

# Unadjusted models included cognitive change scores as the dependent variable, and time and change in NPI-A as independent variables with the interaction term (change in NPI-A * treatment group). Covariates for adjusted models included level of education, age, sex, diabetes, baseline NPI-A, respective baseline cognitive score, and interaction term (change in NPI-A*treatment group).

Abbreviations: MPH, methylphenidate; PLB, placebo; AD, Alzheimer’s Disease; SSRI, selective serotonin reuptake inhibitors; SNRI, serotonin and norepinephrine reuptake inhibitors; NPI, neuropsychiatric inventory; MMSE, Mini Mental State Examination; CF, Category Fluency; HVLT-I, Hopkins Verbal Learning-immediate recall; HVLT-D, Hopkins Verbal Learning-delayed recall; AV, Action Verbal Fluency; BNT, Boston Naming Test; DF, Digit Forward Span; DB, Digit Backward Span; TMT-A, Trail Making Test-A; TMT-B, Trail Making Test-B; HS, high school; df, degrees of freedom.

*** p < 0.005 after Bonferroni correction

## Appendix A. Supporting information

Supplementary data associated with this article can be found in the online version at doi:10.1016/j.inpsyc.2024.100012.
