# Supplementary material for: Correlation between changes in apathy and cognition in Alzheimer’s disease associated apathy: Analysis of the Apathy in Dementia Methylphenidate Trial 2 (ADMET 2)
Source: Int Psychogeriatr. Author manuscript; Available in PMC 2025 Dec 16. (PMC12706841; doi:10.1016/j.inpsyc.2024.100012)
Supplement: Supplementary Table 1 [file NIHMS2127157-supplement-Supplementary_Table_1.docx]

**Supplementary Table 1.** Change in cognitive scores at 2, 4, and 6- month follow-up by treatment group

Summary: This table lists the cognitive change scores at each time point for the methylphenidate and placebo groups

| Measure | **MPH** | | | **PLB** | | |
| --- | --- | --- | --- | --- | --- | --- |
|  | Change score at 2-months | Change score at 4-months | Change score at 6-months | Change score at 2-months | Change score at 4-months | Change score at 6-months |
| **MMSE** | -0.52 (3.06) | -0.83 (2.97) | -1.39 (2.95) | -0.45 (2.50) | -0.73 (2.51) | -1.01 (2.99) |
| **CF** | -0.59 (3.08) | -0.65 (3.48) | -0.95 (3.80) | 0.09 (2.65) | -1.00 (2.66) | -0.36 (3.23) |
| **HVLT- I** | -0.43 (3.14) | -0.77 (3.01) | -0.34 (4.03) | -0.83 (3.05) | -0.52 (3.67) | -0.22 (3.96) |
| **HVLT-D** | 0.01 (1.21) | 0.11 (1.57) | -0.00 (1.36) | -0.14 (1.07) | -0.14 (0.984) | -0.02 (1.14) |
| **AV** | 0.31 (3.71) | 0.80 (3.52) | -0.01 (3.03) | 0.02 (3.39) | -0.29 (2.86) | 0.26 (3.60) |
| **BNT** | -0.51 (2.07) | -0.37 (2.14) | -0.64 (1.77) | -0.51 (1.78) | -0.75 (1.95) | -0.75 (2.06) |
| **DF** | 0.03 (1.87) | 0.29 (1.87) | 0.14 (1.66) | 0.06 (1.40) | -0.01 (1.77) | 0.07 (1.49) |
| **DB** | -0.06 (1.91) | 0.15 (1.95) | 0.29 (1.50) | 0.03 (1.66) | -0.27 (2.22) | -0.18 (1.86) |
| **TMT-A (seconds)** | 11.3 (44.4) | 4.97 (43.6) | 3.90 (33.9) | 0.04 (41.8) | 2.64 (48.8) | 3.17 (45.8) |
| **TMT-B (seconds)** | 11.4 (46.0) | 1.42 (35.4) | 3.86 (47.5) | -4.77 (51.1) | -0.35 (48.3) | -16.9 (49.4) |

Abbreviations: MMSE, Mini Mental State Examination; CF, category fluency HVLT-I, Hopkins Verbal Learning Test- immediate recall; HVLT-D, Hopkins Verbal Learning Test- delayed recall; AV, Action verbal fluency test; BNT, Short Boston Naming test; DF, Digit forward test; DB, Digit backward test; TMTA, Trail Making Test A; TMTB, Trail Making Test B
