# Supplementary material for: Correlation between changes in apathy and cognition in Alzheimer’s disease associated apathy: Analysis of the Apathy in Dementia Methylphenidate Trial 2 (ADMET 2)
Source: Int Psychogeriatr. Author manuscript; Available in PMC 2025 Dec 16. (PMC12706841; doi:10.1016/j.inpsyc.2024.100012)
Supplement: Supplementary Table 2 [file NIHMS2127157-supplement-Supplementary_Table_2.docx]

**Supplementary table 2.** Changes in cognition over time among all participants (n=199) on linear mixed models^#^

Summary: This table lists the cognitive changes over time in all participants.

# Model included the following variables: visit ID, change in NPI-A, treatment, change in NPI-A x treatment, baseline NPI-A, respective baseline cognitive score, age, sex, level of education and diabetes.

Abbreviations: Mini Mental State Examination; CF, Category Fluency; HVLT-I, Hopkins verbal learning- immediate recall; HVLT-D, Hopkins verbal learning- delayed recall; AV, Action verbal fluency; BNT, Boston Naming Test; DF, Digit forward span; DB, Digit backward span; TMT-A, Trail Making Test- A; TMT-B, Trail Making Test-B; df, degrees of freedom.

***** p<0.005** **after Bonferroni correction**

| Cognitive Test | Visit ID | All participants | | | | |
| --- | --- | --- | --- | --- | --- | --- |
|  |  | Unstandardized coefficients | | df | t-statistic | p |
|  |  | B | SE |  |  |  |
| **MMSE** | 2-month | -0.86 | 0.23 | 498.72 | -3.70 | 0.0002*** |
|  | 4-month | -0.97 | 0.24 | 500.53 | -4.07 | 0.00006*** |
|  | 6-month | -1.41 | 0.24 | 500.29 | -5.92 | 0.000000006 *** |
| **CF** | 2-month | -0.60 | 0.27 | 489.97 | -2.22 | 0.03 |
|  | 4-month | -1.08 | 0.28 | 492.50 | -3.91 | 0.0001*** |
|  | 6-month | -1.17 | 0.28 | 493.55 | -4.16 | 0.00004*** |
| **HVLT-I** | 2-month | -1.06 | 0.30 | 495.40 | -3.53 | 0.0005*** |
|  | 4-month | -0.86 | 0.31 | 499.33 | -2.77 | 0.01 |
|  | 6-month | -0.68 | 0.31 | 498.62 | -2.19 | 0.03 |
| **HVLT-D** | 2-month | -0.16 | 0.10 | 472.98 | -1.55 | 0.12 |
|  | 4-month | -0.07 | 0.11 | 476.01 | -0.63 | 0.53 |
|  | 6-month | -0.19 | 0.11 | 474.62 | -1.75 | 0.08 |
| **AV** | 2-month | 0.22 | 0.29 | 485.65 | 0.75 | 0.45 |
|  | 4-month | 0.50 | 0.29 | 489.47 | 1.70 | 0.09 |
|  | 6-month | 0.25 | 0.30 | 490.84 | 0.85 | 0.39 |
| **BNT** | 2-month | -0.47 | 0.18 | 497.46 | -2.67 | 0.01 |
|  | 4-month | -0.48 | 0.18 | 502.55 | -2.63 | 0.01 |
|  | 6-month | -0.73 | 0.18 | 504.09 | -3.98 | 0.00008*** |
| **DF** | 2-month | -0.04 | 0.14 | 495.63 | -0.31 | 0.76 |
|  | 4-month | 0.01 | 0.15 | 499.78 | 0.07 | 0.95 |
|  | 6-month | 0.02 | 0.15 | 499.29 | 0.12 | 0.90 |
| **DB** | 2-month | -0.13 | 0.15 | 489.51 | -0.90 | 0.37 |
|  | 4-month | -0.06 | 0.15 | 492.48 | -0.41 | 0.68 |
|  | 6-month | -0.01 | 0.15 | 491.92 | -0.06 | 0.95 |
| **TMT-A** | 2-month | 8.22 | 4.41 | 402.63 | 1.86 | 0.06 |
|  | 4-month | 7.49 | 4.43 | 401.97 | 1.69 | 0.09 |
|  | 6-month | 6.68 | 4.60 | 404.64 | 1.45 | 0.15 |
| **TMT-B** | 2-month | 6.35 | 7.01 | 159.47 | 0.91 | 0.37 |
|  | 4-month | 2.38 | 6.90 | 153.98 | 0.35 | 0.73 |
|  | 6-month | -2.59 | 7.26 | 161.33 | -0.36 | 0.72 |
