# Supplementary material for: Correlation between changes in apathy and cognition in Alzheimer’s disease associated apathy: Analysis of the Apathy in Dementia Methylphenidate Trial 2 (ADMET 2)
Source: Int Psychogeriatr. Author manuscript; Available in PMC 2025 Dec 16. (PMC12706841; doi:10.1016/j.inpsyc.2024.100012)
Supplement: Supplementary Table 3 [file NIHMS2127157-supplement-Supplementary_Table_3.docx]

**Supplementary table 3.** Change in HVLT- I scores over time in each treatment group in linear mixed models^#^

| Cognitive Test | Visit ID | MPH group | | | | | PLB group | | | | |
| --- | --- | --- | --- | --- | --- | --- | --- | --- | --- | --- | --- |
|  |  | Unstandardized coefficients | | df | t-statistic | p | Unstandardized coefficients | | df | t-statistic | p |
|  |  | B | SE |  |  |  | B | SE |  |  |  |
| **HVLT-I** | 2-month | -0.28 | 0.46 | 237.69 | -0.61 | 0.54 | -1.58 | 0.39 | 252.52 | -4.01 | 0.00008*** |
|  | 4-month | -0.41 | 0.46 | 238.26 | -0.89 | 0.37 | -1.15 | 0.42 | 255.83 | -2.75 | 0.006 |
|  | 6- month | 0.07 | 0.47 | 236.69 | 0.15 | 0.88 | -1.22 | 0.42 | 256.95 | -2.91 | 0.004*** |

Summary: This table lists the verbal learning- immediate recall scores over time for methylphenidate and placebo groups

^#^ Models included the following variables: visit ID, change in NPI-A, baseline NPI-A, respective baseline cognitive score, age, sex, level of education and diabetes

***** p<0.005** **after Bonferroni correction**
